# Comparison of balanced and unbalanced crystalloids as resuscitation fluid in patients treated for cardiogenic shock

**DOI:** 10.1186/s40560-023-00687-y

**Published:** 2023-09-06

**Authors:** Jonas Gmeiner, Bernhardt Bulach, Enzo Lüsebrink, Leonhard Binzenhöfer, Danny Kupka, Thomas Stocker, Kornelia Löw, Ludwig Weckbach, Wolf-Stephan Rudi, Tobias Petzold, Stefan Kääb, Jörg Hausleiter, Christian Hagl, Steffen Massberg, Martin Orban, Clemens Scherer

**Affiliations:** 1 Department of Medicine I, LMU University Hospital, LMU Munich, Marchioninistr. 15, 81377 Munich, Germany; 2 Munich Heart Alliance, German Center for Cardiovascular Research (DZHK), Munich, Germany; 3 Department of Medical Oncology and Hematology, University Hospital Zurich, Zurich, Switzerland; 4 Department of Cardiac Surgery, LMU University Hospital, LMU Munich, Munich, Germany

**Keywords:** Cardiogenic shock, Cardiac arrest, Fluid resuscitation

## Abstract

**Background:**

The efficacy and safety of saline versus balanced crystalloid solutions in ICU-patients remains complicated by exceptionally heterogenous study population in past comparative studies. This study sought to compare saline and balanced crystalloids for fluid resuscitation in patients with cardiogenic shock with or without out-of-hospital cardiac arrest (OHCA).

**Methods:**

We retrospectively analyzed 1032 propensity score matched patients with cardiogenic shock from the Munich University Hospital from 2010 to 2022. In 2018, default resuscitation fluid was changed from 0.9% saline to balanced crystalloids. The primary endpoint was defined as 30-day mortality rate.

**Results:**

Patients in the saline group (*n* = 516) had a similar 30-day mortality rate as patients treated with balanced crystalloids (*n* = 516) (43.1% vs. 43.0%, *p* = 0.833), but a higher incidence of new onset renal replacement therapy (30.2% vs 22.7%, *p* = 0.007) and significantly higher doses of catecholamines. However, OHCA-patients with a lactate level higher than 7.4 mmol/L had a significantly lower 30-day mortality rate when treated with saline (58.6% vs. 79.3%, *p* = 0.013). In addition, use of balanced crystalloids was independently associated with a higher mortality in the multivariate cox regression analysis after OHCA (hazard ratio 1.43, confidence interval: 1.05–1.96, *p* = 0.024).

**Conclusions:**

In patients with cardiogenic shock, use of balanced crystalloids was associated with a similar all-cause mortality at 30 days but a lower rate of new onset of renal replacement therapy. In the subgroup of patients after OHCA with severe shock, use of balanced crystalloids was associated with a higher mortality than saline.

*Trial registration*: LMUshock registry (WHO International Clinical Trials Registry Platform Number DRKS00015860).

**Supplementary Information:**

The online version contains supplementary material available at 10.1186/s40560-023-00687-y.

## Background

Fluid resuscitation is one of the most frequently used treatment for critically ill patients in the intensive care unit. Even though practiced since the nineteenth century, larger trials comparing different fluids have been absent until two decades ago [1]. Since then, albumin has been shown to increase mortality among patients suffering from traumatic brain injury [2, 3]. Another colloid, hydroxyethyl starch, has been found to increase the risk of acute kidney injury and death, leaving intensive care physicians with crystalloid fluids to choose from [4, 5]. Saline (0.9% sodium chloride) is the most commonly used crystalloid, but its use was associated with a higher risk of hyperchloremic acidosis, renal failure and in some studies mortality compared to balanced solutions that have a chloride concentration closer to that of blood plasma [6–10]. As subsequent randomized controlled trials failed to reproduce these findings, there is ongoing debate on which crystalloid fluid to use with the latest meta-analysis reporting a high probability that balanced crystalloids reduce mortality compared to saline [1, 6, 11]. However, available evidence is limited to a heterogenic study population with a high prevalence of postoperative patients ranging up to 71% in some studies, whereas the amount of non-operative cardiovascular admissions is either not reported or as low as 5% [6, 9]. Additionally, balanced electrolytes were associated with a higher mortality than saline in patients after traumatic brain injury, but data for patients after non-traumatic brain injury is lacking.

Cardiogenic shock (CS) patients in particular are not only significantly underrepresented in these trials, but have a very high risk of death with a 30-day mortality rate of around 40–50% and a risk of acute kidney injury requiring renal replacement therapy of up to 21% despite latest advances in treatment such as mechanical circulatory support [12–14]. Furthermore, these patients commonly require large volumes of intravenous fluids in addition to high doses of catecholamines to maintain sufficient circulation, making them potentially more susceptible to even smaller treatment effects of different fluids. To date, no study has compared different fluid resuscitation strategies in CS patients and specific guideline recommendations are lacking. To the best of our knowledge this is the first study comparing saline and balanced electrolytes in patients with CS with or without out-of-hospital cardiac arrest (OHCA). The primary hypothesis was that the use of balanced electrolytes reduces 30-day all-cause mortality rate in patients with CS.

## Methods

### Study population

All CS patients treated in the cardiac intensive care unit (ICU) of the Ludwig-Maximilians-University hospital between January 2010 and August 2022 were included in the LMUshock registry (WHO International Clinical Trials Registry Platform Number DRKS00015860). Data collection and analysis was in accordance with the Declaration of Helsinki and German data protection laws. The study was approved by the local ethics committee (IRB number: 18–001). CS was defined by ESC guidelines, the IABP-SHOCK II trial and CULPRIT SHOCK trial [13–15]. Telephone follow-up was performed for patients dismissed from the hospital before day 30.

### Intravenous fluids

From 2018 on, the default maintenance and resuscitation fluid in our institution was switched from saline (0,9% sodium chloride, B. Braun SE, Melsungen, Germany) to a balanced resuscitation fluid (Jonosteril®, Fresenius Kabi, Bad Homburg, Germany). Jonosteril® is a balanced crystalloid buffered with acetate that has electrolyte concentrations closer to those of blood plasma than saline (Jonosteril®: Na + 137 mmol/l, Cl − 110 mmol/l, K + 4 mmol/l, Ca2 + 1.65 mmol/l, Mg2 + 1.25 mmol/l, acetate 36.8 mmol; Saline: Na + 154 mmol/l, Cl− 154 mmol/l). This decision was driven by the results of the SMART trial and was upheld since then [8]. However, a different choice of resuscitation fluid was allowed at the discretion of the attending intensive care physician. Patients receiving Jonosteril® at the day of admission to ICU were assigned to the balanced group, while patients receiving exclusively unbalanced fluid as resuscitation fluid were assigned to the saline group. Fluids required for dilution of parenteral medication were not analyzed due to missing data. Output data was available as total fluid output including urine, feces and iatrogenic output such as pleuracentesis or dialysis.

### Study endpoints and parameters

The primary endpoint was defined as all-cause 30-day mortality rate. Secondary endpoints included time on ICU, serum creatinine, new onset of renal-replacement therapy, time-weighted catecholamine doses (using the formula: dobutamine (mg/h) + 100 × epinephrine (mg/h) + 100 × norepinephrine (mg/h) as previously described), blood pressure, arterial blood pH, bicarbonate, serum chloride concentration [16]. In patients after OHCA, peak neuron specific enolase (NSE) within 3 days after resuscitation was assessed as a predictor of neurologic outcome in resuscitated patients [17].

### Statistical analysis

Statistical analysis was performed in accordance with the STROBE (Strengthening the Reporting of Observational Studies in Epidemiology) statement using the software R (version 4.2.2, The R foundation, Vienna, Austria) [18]. Normally distributed continuous variables were depicted as mean with standard deviation and non-normally distributed continuous variables as median with interquartile ranges. Students *t*-test and Mann–Whitney-*U* test were used to compare groups as appropriate. Categorical variables were reported as absolute numbers and percentages and Chi-squared test was utilized for comparison. All tests were 2-tailed, and *p*-values < 0.05 were considered significant. Mortality rates were calculated using the Kaplan–Meier method and comparisons were made by log-rank tests. Missing data for body mass index and Simplified Acute Physiology Score 2 was imputed by R package “mice” (version 3.13.0) with predictive mean matching. Univariate and multivariate cox regression analysis was performed to identify predictors for the primary endpoint of 30-day all-cause mortality. Parameters for multivariate analysis were stepwise selected by Akaike information criterion (AIC) with backward direction and 1000 bootstrap iterations using the stepAIC function of the R-package MASS (version 7.3–58.1).

R-package MatchIt (version 4.5.0; Ho, Imai, King, and Stuart) was used for propensity score matching with a 1:1 nearest neighbour algorithm, no replacement, a 0.1 caliper and the following variables:[19]. age, sex, estimated glomerular filtration rate, OHCA, duration of out-of-hospital cardiopulmonary resuscitation, myocardial infarction (STEMI, NSTEMI) as well as mechanical ventilation, VA-ECMO, percutaneous transvalvular microaxial flow pump (Impella®), catecholamine doses and serum lactate at admission. Subgroup analyses were conducted for patients after OHCA as well as for patients receiving more than the median hourly volume of resuscitation fluids during the first day. The subgroup of OHCA patients was then divided into two groups based on the median serum lactate at admission to assess OHCA patients with severe shock.

## Results

### Baseline characteristics

All consecutive CS patients from 2010 to 2022 were included in the analysis (Additional file 1: Fig. S1). After propensity score matching groups were well balanced with a standard difference of mean below 0.15 for all matching parameters. (Additional file 1: Fig. S2). Distribution of treatment years after matching is displayed in Additional file 1: Table S1. Characteristics of the 1032 matched patients are shown in Table 1. Patients in the saline group had less often undergone coronary artery bypass grafting and were more frequently on intraaortic ballon pump. On admission to the ICU serum lactate, arterial blood pH, bicarbonate concentration, serum creatinine, chronic dialysis and serum chloride did not differ between groups. Most patients were admitted for CS complicating myocardial infarction (23.7% NSTEMI, 29.3% STEMI, Table 1). Patients were presenting with a high percentage of OHCA (25.4% vs. 24.8%, *p* = 0.886) and venoarterial extracorporeal membrane oxygenation (22.5% vs. 23.1%, *p* = 0.882).

**Table 1:** Patient characteristics after propensity score matching

	Saline (*n* = 516)	Balanced crystalloids (*n* = 516)	*p*-value
*Past medical history*			
Age	65.7 ± 15.1	65.8 ± 14.9	0.929
Male sex	73.4%	72.7%	0.833
Smoker			0.213
Active smoker	22.5%	22.1%	
Former smoker	21.5%	17.4%	
Never smoked	56.0%	60.5%	
Hypertension	72.5%	72.1%	0.945
Dyslipidemia	56.2%	54.3%	0.573
Diabetes mellitus	33.7%	34.9%	0.743
Previous myocardial infarction	25.2%	23.1%	0.467
Previous PCI	32.0%	33.5%	0.642
Previous CABG	8.7%	13.4%	**0.022**
Previous stroke	11.0%	8.7%	0.251
Peripheral arterial disease	13.0%	13.2%	1.000
Chronic dialysis	2.7%	1.9%	0.536
*Status on admission*			
Type of CS			0.997
STEMI	29.3%	29.3%	
NSTEMI	23.6%	23.8%	
Non-ACS	47.1%	46.9%	
Primary PCI	53.7%	56.6%	0.381
Contrast agent (ml) if angiography was performed	174.3 ± 155.6	177.2 ± 132.6	0.782
Out of hospital cardiac arrest	25.4%	24.8%	0.886
Duration of CPR (min)	13.0 (0.0–26.0)	12.0 (0.0–26.0)	0.813
Mechanical ventilation	54.7%	55.0%	0.950
Hypothermia (< 35.0 °C)	23.1%	21.9%	0.709
VA-ECMO	22.5%	23.1%	0.882
Percutaneous transvalvular microaxial flow pump (Impella®)	6.4%	6.6%	1.000
SAVE score	− 8.9 ± 5.3	− 8.7 ± 5.3	0.731
IABP	8.3%	0.2%	** < 0.001**
Catecholamines			
Norepinephrine (mg/h)	0.8 ± 1.8	0.9 ± 1.9	0.395
Dobutamine (mg/h)	2.7 ± 8.5	2.2 ± 5.7	0.251
Epinephrine (mg/h)	0.7 ± 3.2	0.4 ± 1.3	**0.016**
Ejection fraction	30.0 (20.0–43.0)	30.0 (20.0–45.0)	0.343
eGFR	46.0 (30.0–60.0)	46.0 (28.8–65.0)	0.210
Serum lactate	4.7 (2.0–9.0)	4.4 (2.3–8.4)	0.966
Serum chloride	106.2 ± 7.1	105.6 ± 6.9	0.186
Serum sodium	137.0 ± 8.8	138.1 ± 8.8	0.065
Serum potassium	4.2 ± 0.8	4.3 ± 0.8	0.058
Serum osmolality	287.7 ± 18.2	288.1 ± 18.0	0.706
SAPS II	76.0 (67.0–83.0)	75.0 (67.0–81.0)	0.172
*Post admission*			
New onset renal replacement therapy	30.2%	22.7%	**0.007**
CPR during ICU stay	18.2%	13.4%	**0.041**
Duration of ICU stay (days)	5.6 (1.9–12.5)	5.1 (1.9–10.1)	0.358
Duration of VA-ECMO (days)	3.8 ± 2.5	4.6 ± 4.3	0.053

Values are depicted as no. (percentage of total no.), mean +—standard deviation or median (interquartile range) as appropriate

*BMI* body mass index, *CABG* coronary artery bypass graft, *CPR* cardiopulmonary resuscitation, *eGFR* estimated glomerular filtration rate, *IABP* intraaortic ballon pump, *ICU* intensive care unit, *PCI* percutaneous coronary intervention, *SAPS II* Simplified Acute Physiology Score II, *SAVE score* Survival after Veno-Arterial ECMO Score, *VA-ECMO* venoarterial extracorporeal membrane oxygenation

### Fluids and electrolytes

**Fig. 1:**
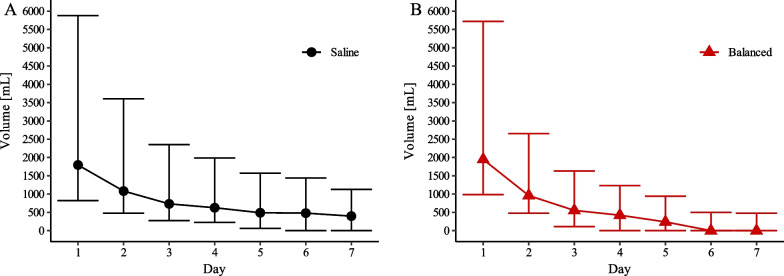
Resuscitation fluid volumes. Median volumes (quartiles) of saline and balanced crystalloids administered as resuscitation fluid within the first 7 days of intensive care unit stay in the saline group (**A**) and balanced crystalloids group (**B**)

**Fig. 2:**
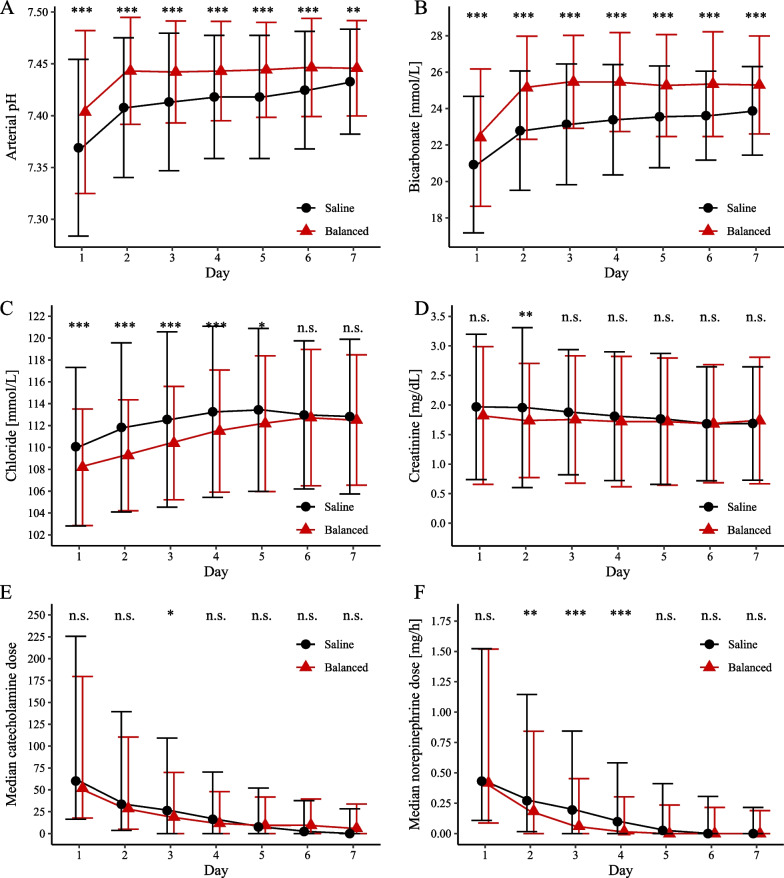
Arterial blood pH, bicarbonate, chloride, creatinine concentration and catecholamine doses. Mean values (standard deviation) of arterial blood pH (**A**), bicarbonate (**B**), chloride concentration (**C**) and creatinine (**D**) as well as median catecholamine doses (**E**, using the formula: dobutamine (mg/h) + 100 × epinephrine (mg/h) + 100 × norepinephrine (mg/h)) and norepinephrine (**F**) in patients treated with saline (Saline, black) and balanced crystalloids (Balanced, red). *n.s.* not significant. *p < 0.05; **p < 0.01; ***p < 0.001

Volumes of saline and balanced crystalloids administered as resuscitation fluid within the first seven days on the ICU are shown in Fig. 1. Cumulative fluid volume within the first week was comparable between the saline and balanced crystalloid group (mean volume in milliliters ± standard deviation: 8436.7 ± 9227.8 vs. 9086.3 ± 10,690.7, *p* = 0.296). Patients treated with saline had significantly higher levels of serum chloride and lower levels of bicarbonate as well as arterial blood pH (Fig. 2). Serum creatinine was comparable during the first seven days except for a significantly higher value in the saline group at day 2 (Fig. 2). Total fluid output was similar between groups on the first 7 days on ICU.

### Outcomes

**Fig. 3:**
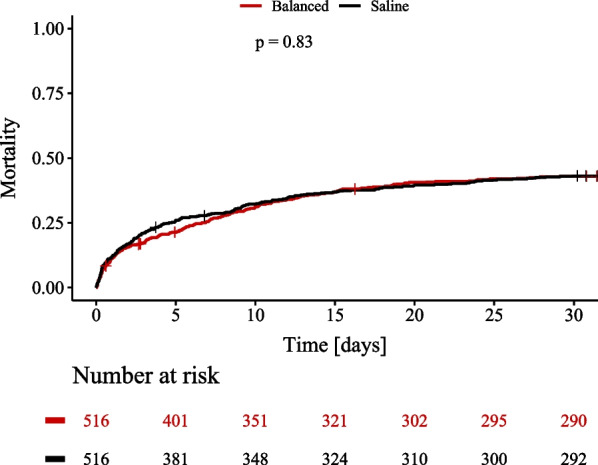
30-day all-cause mortality rate. Kaplan–Meier curves of the primary endpoint of 30-day all-cause mortality for patients treated with saline (Saline, black) and balanced crystalloids (Balanced, red)

Cumulative catecholamine doses within the first week in ICU are shown in Fig. 2. Combined catecholamine doses were numerically higher in the saline group during the first days with a significant difference on day 3 (Fig. 2E). Noradrenaline doses were significantly higher in the saline group at day two to four (Fig. 2F) despite significantly lower blood pressure in the saline group (Additional file 1: Table S3). The 30-day mortality rate was 43.1% in the saline group vs. 43.0% in the balanced crystalloid group (*p* = 0.833, Fig. 3). In addition, use of balanced crystalloids was not significantly associated with mortality in a cox regression analysis that included CS patients before matching (Additional file 1: Table S2). Length of stay in ICU did not differ between groups (median days (interquartile range): 5.6 (1.9–12.5) vs 5.1 (1.9–10.1) days, *p* = 0.358). Patients in the saline group had a higher rate of new onset renal replacement therapy (30.2% vs 22.7%, *p* = 0.007) as well as a higher rate of in-hospital cardiopulmonary resuscitation (18.2% vs. 13.4%, *p* = 0.041).

### Subgroup analyses

**Fig. 4:**
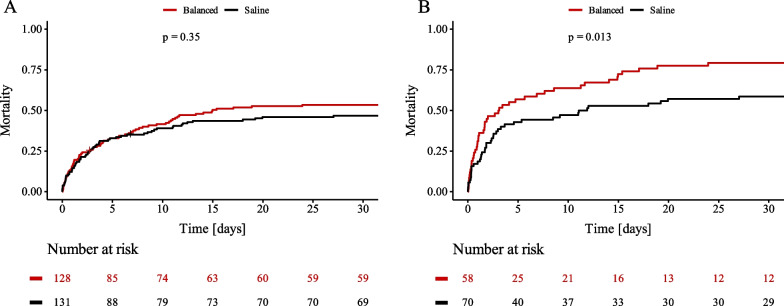
30-day all-cause mortality rate of patients after out-of-hospital cardiac arrest. Kaplan–Meier curves showing 30-day all-cause mortality for patients after out-of-hospital cardiac arrest treated with saline (black) and balanced crystalloids (Balanced, red) (**A**) and for patients after out-of-hospital cardiac arrest with lactate at admission > 7.4 mmol/L (median) (**B**)

Patients after OHCA (Additional file 1: Table S4) showed a trend towards higher 30-day mortality rate when treated with balanced crystalloids group that did not reach statistical significance (53.2% vs 46.7%, *p* = 0.356, Fig. 4A). Patients with OHCA with lactate levels higher than 7.4 mmol/L (median of the former group) showed a higher mortality rate (79.3% vs 58.6%, *p* = 0.013, Fig. 4B) in the balanced group. In these patients, NSE was comparable at admission but significantly higher at 48–72 h after admission when treated with balanced crystalloids (104.6 (60.0–286.0) vs. 67.9 (34.6–148.2), *p* = 0.039). Furthermore, use of balanced crystalloids as resuscitation fluid as well as age, serum lactate, duration of CPR and mechanical ventilation were independently associated with a higher 30-day mortality rate in a multivariate analysis including all patients with OHCA before propensity-score matching (Table 2). Patients without OHCA showed a comparable 30-day all-cause mortality rate when treated with saline or balanced electrolytes (41.9% vs. 39.5%, *p* = 0.427).

**Table 2:** Cox regression analysis in patients with out-of-hospital cardiac arrest

	Univariate			Multivariate		
	Hazard ratio	CI	*p*-value	Hazard ratio	CI	*p*-value
Age (years)	1.03	1.01–1.04	** < 0.001**	1.03	1.02–1.05	** < 0.001**
Male gender	0.99	0.68–1.44	0.963			
Lactate at admission (mmol/l)	1.17	1.13–1.20	** < 0.001**	1.14	1.10–1.18	** < 0.001**
NSTEMI	1.28	0.86–1.90	0.219			
STEMI	1.07	0.75–1.52	0.709			
Cardiac arrest	1.36	0.19–9.71	0.760			
Duration of OHCA CPR (min)	1.02	1.01–1.02	** < 0.001**	1.01	1.00–1.02	**0.004**
Catecholamine dose at admission (au/h)	1.00	1.00–1.00	** < 0.001**			
eGFR (ml/min/1,73m^2^)	0.98	0.97–0.99	** < 0.001**	0.99	0.98–1.00	0.078
VA-ECMO at admission	1.52	1.12–2.08	**0.008**			
Percutaneous transvalvular microaxial flow pump (Impella) at admission	1.47	0.81–2.65	0.197			
Mechanical ventilation at admission	14.76	2.07–105.43	**0.007**	8.36	1.16–60.16	**0.035**
Use of balanced crystalloids	1.04	0.77–1.41	0.806	1.43	1.05—1.96	**0.024**

Univariate and multivariate cox-regression analysis regarding 30-day all-cause mortality rate in all unmatched patients with out-of-hospital cardiac arrest

*au* arbitrary units, *Cardiac arrest* cardiac arrest before or during ICU stay, *eGFR* estimated glomerular filtration rate, *NSTEMI* non-ST-elevation myocardial infarction, *OHCA* out-of-hospital cardiac arrest, *CPR* cardiopulmonary resuscitation, *STEMI* ST-elevation myocardial infarction, *VA-ECMO* venoarterial extracorporeal membrane oxygenation

In another subgroup analysis the study cohort was divided into two subgroups each based on the median hourly volume of resuscitation fluid during the first day as well as the median serum lactate at admission. Among patients receiving more than median fluid volume (Additional file 1: Table S5), mortality rate at 30 days did not differ significantly between patients treated with saline and balanced crystalloids (51.2% vs 54.5%, *p* = 0.602). Among patients with higher than median serum lactate at admission, mortality rate at 30 days did not differ between groups (59.0% vs. 59.2%, *p* = 0.844).

## Discussion

In this retrospective single center study comparing saline and balanced crystalloids as resuscitation fluid for CS patients, we did not find a significantly different mortality at 30 days, but a higher rate of new onset renal replacement therapy and higher doses of catecholamines among patients treated with saline.

Studies comparing the efficacy and safety of saline versus balanced resuscitation fluids produced conflicting results [1, 6, 8, 9, 11, 20]. While some studies found significantly higher rates of mortality and kidney failure among ICU patients treated with saline, newer randomized trials failed to reproduce the observed advantages of balanced crystalloids. Our trial is in concordance with published results showing no impact of saline versus crystalloid solution on overall 30-day mortality. However, our study is very distinct from the available evidence. While most of the preceding studies focused on unselected ICU patients with a high percentage of (elective) postoperative and sepsis patients, our study analysis the specific patient population with CS admitted to a tertiary center cardiac ICU. Compared to a heterogenous “all-comer” ICU collective, CS patients are severely ill with a very high risk of mortality and multiorgan failure including acute kidney failure. As such, baseline levels of serum lactate were much higher in our study cohort compared to patients in the PLUS trial, the only randomized trial even reporting baseline lactate [11]. CS patients typically need large amounts of resuscitation fluids to maintain sufficient circulation and when mechanical circulatory devices are implanted. Thus, cumulative volume of intravenous fluid in our study exceeded that of most preceding studies. Additionally, more than one third of patients required mechanical circulatory support, increasing the need for intravenous fluids even further, and rates of cardiac arrest were much higher in our patient population.

In previous studies, saline has been shown to cause hyperchloremic acidosis due to its high concentration of chloride [20]. Consistently, we found significantly higher levels of serum chloride and a significantly lower pH in the saline group. This hyperchloremic acidosis might explain the need for higher catecholamine doses that we found in the saline group through a direct effect on blood pressure and attenuation of catecholamines as well as the higher rate of new onset renal replacement therapy in the saline group [21, 22].

In our study, the primary endpoint of mortality rate at 30 days did not differ between groups and results remained unchanged when limiting the analysis to patients receiving large amounts of resuscitation fluids. This could be explained with the poor prognosis of CS patients. Even if balanced crystalloids might decrease acidosis and catecholamine demand in the early stages of CS, potential treatment effects are masked by the high mortality and unfavorable neurological outcome after cardiac arrest leading to palliative care.

In the BaSICS study, balanced crystalloids were associated with an increased 90-day mortality in patients after traumatic brain injury [9]. As these patients often develop intracranial hypertension, use of iso- or even hypertonic saline is often preferred over balanced electrolytes which are relatively hypotonic. In our study, 25% of patients experienced OHCA with a median duration of cardiopulmonary resuscitation of 12 min, indicating significant hypoxic brain injury. For patients after OHCA with a more severe shock state (lactate > median of OHCA cohort) we could demonstrate a significantly higher 30-day mortality rate in the balanced electrolytes group and a multivariate regression analysis revealed balanced electrolytes as an independent risk factor for mortality. Additionally, NSE was higher in the OHCA group with severe shock when treated with balanced crystalloids. These findings raise concerns that balanced fluids might be harmful in patients after non-traumatic brain injury. In accordance with results from patients with traumatic brain injury saline might be used as preferred resuscitation fluid in patients after OHCA with a more severe shock state until further evidence is provided from randomized controlled trials [9].

### Limitations

This study is a retrospective single center analysis of patients from the LMUshock registry with its inherent limitations. Fluids administered before hospital admission and in the emergency department before admission to ICU as well as fluids for dilution of parenteral medications are not included in the analysis due to missing data. In our study, one specific balanced crystalloid solution was used and results might differ with other solutions, e.g. ringer’s lactate. Time of inclusion differed between the two groups by a few years and there might be differences in treatments, such as intraaortic balloon pump or indications for renal replacement therapy, not accounted for by the propensity score matching. Lastly, CS patients represent a unique patient population and findings should not be generalized. Subgroup analyses are hypothesis generating only and should be interpreted as such.

## Conclusions

In this retrospective study of cardiogenic shock patients, treatment with balanced crystalloids as resuscitation fluid was associated with a similar all-cause mortality at 30 days but a lower incidence of renal replacement therapy than treatment with unbalanced crystalloids. In the subgroup of patients after out-of-hospital cardiac arrest with severe shock, use of balanced crystalloids was associated with a higher 30-day mortality rate.

### Supplementary Information

**Additional file 1: Figure S1.** Study flowchart. **Figure S2.** Standardized mean differences before and after propensity score matching. Love plot depicting standardized mean differences before (unadjusted) and after propensity score matching (adjusted). **Table S1.** Year of inclusion. **Table S2.** Cox regression analysis. **Table S3.** Blood pressure. **Table S4.** Subgroup characteristics for patients after out-of-hospital cardiac arrest. **Table S5.** Subgroup characteristics for patients that received high amounts of resuscitation fluid.

## Data Availability

The datasets generated and/or analysed during the current study are not publicly available due to ethical committee regulations but might be available from the corresponding author on reasonable request and provided that ethical committee approves data sharing.

## Abbreviations

CS: Cardiogenic shock
ICU: Intensive care unit
NSTEMO: Non-ST-elevation myocardial infarction
OHCA: Out-of-hospital cardiac arrest
STEMI: ST-elevation myocardial infarction
STROBE: Strengthening the Reporting of Observational Studies in Epidemiology
VA-ECMO: Venoarterial extracorporeal membrane oxygenation
