# Supplementary material for: Comparison of balanced and unbalanced crystalloids as resuscitation fluid in patients treated for cardiogenic shock
Source: J Intensive Care. 2023 Sep 6;11:38. doi: 10.1186/s40560-023-00687-y (PMC10481512; doi:10.1186/s40560-023-00687-y)
Supplement: Supplementary file 1 — Additional file 1: Figure S1. Study flowchart. Figure S2. Standardized mean differences before and after propensity score matching. Love plot depicting standardized mean differences before (unadjusted) and after propensity score matching (adjusted). Table S1. Year of inclusion. Table S2. Cox regression analysis. Table S3. Blood pressure. Table S4. Subgroup characteristics for patients after out-of-hospital cardiac arrest. Table S5. Subgroup characteristics for patients that received high amounts of resuscitation fluid. [file 40560_2023_687_MOESM1_ESM.docx]

Figure S1: study flowchart

Figure S2: Standardized mean differences before and after propensity score matching

Love plot depicting standardized mean differences before (unadjusted) and after propensity score matching (adjusted).

Table S1: Year of inclusion

| Year of inclusion | Saline (n=516) | Balanced (n=516) |
| --- | --- | --- |
| 2010 | 4 | 0 |
| 2011 | 15 | 0 |
| 2012 | 28 | 0 |
| 2013 | 59 | 3 |
| 2014 | 39 | 1 |
| 2015 | 43 | 3 |
| 2016 | 121 | 3 |
| 2017 | 167 | 1 |
| 2018 | 22 | 100 |
| 2019 | 3 | 90 |
| 2020 | 8 | 120 |
| 2021 | 6 | 133 |
| 2022 | 1 | 62 |

Table S2: Cox regression analysis

|  | Univariate | | | Multivariate | | |
| --- | --- | --- | --- | --- | --- | --- |
|  | Hazard ratio | CI | p-value | Hazard ratio | CI | p-value |
| Age | 1.02 | 1.01-1.02 | **<0.001** | 1.02 | 1.02-1.03 | **<0.001** |
| Male gender | 0.87 | 0.72-1.05 | 0.134 | 0.82 | 0.68-0.99 | **0.041** |
| Lactate ad admission | 1.12 | 1.11-1.13 | **<0.001** | 1.11 | 1.09-1.12 | **<0.001** |
| NSTEMI | 1.67 | 1.35-2.05 | **<0.001** |  |  |  |
| STEMI | 1.46 | 1.20-1.78 | **<0.001** |  |  |  |
| Cardiac arrest | 2.14 | 1.79-2.57 | **<0.001** | 1.59 | 1.281.97 | **<0.001** |
| OHCA | 1.45 | 1.21-1.73 | **<0.001** |  |  |  |
| Duration OHCA CPR | 1.02 | 1.01-1.02 | **<0.001** | 1.01 | 1.01-1.01 | **<0.001** |
| Catecholamine dose at admission | 1.00 | 1.00-1.00 | **0.006** | 1.00 | 1.00-1.00 | **0.018** |
| eGFR | 0.99 | 0.98-0.99 | **<0.001** | 0.99 | 0.99-0.99 | **<0.001** |
| VA-ECMO at admission | 1.77 | 1.47-2.13 | **<0.001** |  |  |  |
| Percutaneous transvalvular microaxial flow pump (Impella) at admission | 1.38 | 1.02-1.86 | **0.034** | 1.51 | 1.11-2.04 | **0.008** |
| Mechanical ventilation at admission | 1.98 | 1.67-2.37 | **<0.001** | 1.18 | 0.95-1.46 | 0.139 |
| Use of balanced crystalloids | 0.82 | 0.69-0.97 | **0.024** |  |  |  |

Univariate and multivariate cox-regression analysis for the primary endpoint of 30-day all-cause mortality in all patients before propensity score matching (n=1287).

Cardiac arrest = cardiac arrest before or during ICU stay

eGFR = estimated glomerular filtration rate

NSTEMI = non-ST-elevation myocardial infarction

OHCA = out-of-hospital cardiac arrest

CPR = cardiopulmonary resuscitation

STEMI = ST-elevation myocardial infarction

VA-ECMO = venoarterial extracorporeal membrane oxygenation

Table S3: Blood pressure

|  | Saline (n=516) | Balanced (n=516) | p-value |
| --- | --- | --- | --- |
| **Systolic** |  |  |  |
| Admission | 83.7 ± 19.8 | 85.0 ± 20.4 | 0.290 |
| Day 1 | 100.9 ± 16.8 | 101.5 ± 16.6 | 0.583 |
| Day 2 | 102.1 ± 15.4 | 105.8 ± 16.4 | **0.001** |
| Day 3 | 105.4 ± 16.5 | 110.4 ± 18.0 | **<0.001** |
| Day 4 | 108.3 ± 16.9 | 112.4 ± 19.8 | **0.004** |
| Day 5 | 112.2 ± 17.5 | 114.6 ± 21.3 | 0.134 |
| Day 6 | 113.4 ± 17.3 | 114.4 ± 21.8 | 0.526 |
| Day 7 | 115.2 ± 17.5 | 113.9 ± 22.2 | 0.483 |
| **Diastolic** |  |  |  |
| Admission | 45.7 ± 12.9 | 47.0 ± 12.3 | 0.110 |
| Day 1 | 56.6 ± 10.9 | 56.9 ± 9.2 | 0.694 |
| Day 2 | 55.7 ± 8.8 | 56.9 ± 8.3 | **0.049** |
| Day 3 | 56.3 ± 8.4 | 59.4 ± 9.5 | **<0.001** |
| Day 4 | 57.4 ± 9.0 | 60.2 ± 9.6 | **<0.001** |
| Day 5 | 58.1 ± 8.6 | 61.1 ± 9.7 | **<0.001** |
| Day 6 | 58.2 ± 8.5 | 60.6 ± 9.7 | **0.002** |
| Day 7 | 58.2 ± 8.2 | 59.8 ± 9.3 | **0.045** |

Mean systolic and diastolic blood pressure during the first week.

Table S4: Subgroup characteristics for patients after out-of-hospital cardiac arrest

|  | Saline (n=131) | Balanced crystalloids (n=128) | p-value |
| --- | --- | --- | --- |
| **Past medical history** |  |  |  |
| Age | 64.8 ± 13.6 | 62.1 ± 15.3 | 0.127 |
| Male sex | 78.6% | 82.8% | 0.486 |
| Smoker |  |  | 0.436 |
| Active smoker | 21.4% | 26.6% |  |
| Former smoker | 15.3% | 10.9% |  |
| Never smoked | 63.4% | 62.5% |  |
| Hypertension | 71.0% | 69.5% | 0.903 |
| Dyslipidemia | 54.2% | 49.2% | 0.498 |
| Diabetes mellitus | 27.5% | 30.5% | 0.694 |
| Previous myocardial infarction | 23.7% | 18.0% | 0.330 |
| Previous PCI | 28.2% | 24.2% | 0.552 |
| Previous CABG | 6.9% | 9.4% | 0.610 |
| Previous stroke | 8.4% | 8.6% | 1 |
| Peripheral arterial disease | 7.6% | 6.2% | 0.847 |
| Chronic dialysis | 0.0% | 1.6% | 0.468 |
| **Status on admission** |  |  |  |
| Type of CS |  |  | 0.530 |
| STEMI | 42.7% | 46.9% |  |
| NSTEMI | 31.3% | 25.0% |  |
| Non-ACS | 26.0% | 28.1% |  |
| Duration of CPR (min) | 20.0 (12.0-33.0) | 16.0 (10.0-35.0) | 0.321 |
| Mechanical ventilation | 96.2% | 92.2% | 0.267 |
| VA-ECMO | 42.7% | 32.8% | 0.128 |
| Percutaneous transvalvular microaxial flow pump (Impella®) | 6.1% | 6.2% | 1.0 |
| SAVE score | -9.5 ± 4.9 | -11.1 ± 4.3 | 0.104 |
| IABP | 6.9% | 0.8% | **0.026** |
| eGFR | 54.0 (43.0-60.0) | 57.0 (42.0-69.3) | 0.465 |
| Serum lactate | 7.9 (5.2-9.7) | 7.1 (3.5-9.4) | 0.138 |
| Serum chloride | 107.0 (103.3- 112.0) | 107.0 (103.0-109.0) | 0.266 |
| Serum sodium | 139.0 (136.0-142.0) | 139.0 (136.0-142.0) | 0.966 |
| Serum potassium | 3.8 (3.3-4.1) | 4.0 (3.5-4.5) | **0.003** |
| Serum osmolality | 290.1 ± 12.3 | 288.3 ± 11.6 | 0.219 |
| SAPS II | 79.0 (73.0-86.0) | 75.0 (67.0-83.0) | **0.012** |
| **Post admission** |  |  |  |
| New onset renal replacement therapy | 19.8% | 13.3% | 0.210 |
| CPR during ICU stay | 13.0% | 11.7% | 0.905 |
| Duration of ICU stay (days) | 8.3 (2.5-13.1) | 6.4 (2.2-11.5) | 0.291 |
| Duration of VA-ECMO (days) | 3.0 ± 1.9 | 3.2 ± 3.0 | 0.687 |

Values are depicted as no. (percentage of total no.), mean +- standard deviation or median (interquartile range) as appropriate.

BMI = body mass index

CABG = coronary artery bypass graft

CPR = cardiopulmonary resuscitation

eGFR = estimated glomerular filtration rate

IABP = intraaortic ballon pump

ICU = intensive care unit

PCI = percutaneous coronary intervention

SAPS II = Simplified Acute Physiology Score II

SAVE score = Survival after Veno-Arterial ECMO Score

VA-ECMO = venoarterial extracorporeal membrane oxygenation

Table S5: Subgroup characteristics for patients that received high amounts of resuscitation fluid

|  | Saline (n=260) | Balanced crystalloids (n=258) | p-value |
| --- | --- | --- | --- |
| **Past medical history** |  |  |  |
| Age | 64.9 ± 14.1 | 65.0 ± 14.7 | 0.925 |
| Male sex | 71.5% | 74.8% | 0.459 |
| Smoker |  |  | 0.789 |
| Active smoker | 24.6% | 25.6% |  |
| Former smoker | 16.9% | 14.7% |  |
| Never smoked | 58.5% | 59.7% |  |
| Hypertension | 73.8% | 74.0% | 1.0 |
| Dyslipidemia | 56.2% | 56.2% | 1.0 |
| Diabetes mellitus | 33.8% | 35.3% | 0.804 |
| Previous myocardial infarction | 25.4% | 21.7% | 0.377 |
| Previous PCI | 31.5% | 32.2% | 0.952 |
| Previous CABG | 7.7% | 13.2% | 0.058 |
| Previous stroke | 10.0% | 8.1% | 0.559 |
| Peripheral arterial disease | 11.2% | 10.9% | 1.0 |
| Chronic dialysis | 0.4% | 1.6% | 0.364 |
| **Status on admission** |  |  |  |
| Type of CS |  |  | 0.497 |
| STEMI | 38.1% | 34.5% |  |
| NSTEMI | 27.7% | 26.4% |  |
| Non-ACS | 34.2% | 39.1% |  |
| OHCA | 36.9% | 34.1% | 0.564 |
| Duration of CPR (min) | 15.0 (0.0-30.0) | 14.0 (0.0-30.0) | 0.959 |
| Mechanical ventilation | 97.6% | 76.7% | 0.493 |
| VA-ECMO |  |  |  |
| Percutaneous transvalvular microaxial flow pump (Impella®) |  |  |  |
| SAVE score | -9.2 ± 5.0 | -9.6 ± 4.3 | 0.538 |
| IABP | 6.9% | 0.4% | **<0.001** |
| eGFR | 49.0 (38.0-60.0) | 51.0 (34.0-66.0) | 0.411 |
| Serum lactate | 8.1 (3.7-9.7) | 7.0 (3.4-9.6) | 0.203 |
| Serum chloride | 107.0 (104.0-111.0) | 107.0 (103.0-110.0) | 0.177 |
| Serum sodium | 138.0 (135.0-142.0) | 139.0 (135.0-142.0) | 0.383 |
| Serum potassium | 4.0 (3.4-4.6) | 4.1 (3.6-4.7) | **0.012** |
| Serum osmolality | 290.3 ± 13.2 | 287.8 ± 14.8 | **0.045** |
| SAPS II | 77.0 (70.0-86.0) | 77.0 (70.0-83.0) | 0.121 |
| **Post admission** |  |  |  |
| New onset renal replacement therapy | 33.5% | 27.1% | 0.141 |
| CPR during ICU stay | 24.6% | 14.7% | **0.007** |
| Duration of ICU stay (days) | 7.0 (1.5-13.9) | 5.6 (1.3-11.6) | 0.221 |
| Duration of VA-ECMO (days) | 3.6 ± 2.5 | 4.6 ± 4.5 | **0.036** |

Subgroup characteristics of patients receiving more than median of the resuscitation fluid volumes. Values are depicted as no. (percentage of total no.), mean +- standard deviation or median (interquartile range) as appropriate.

BMI = body mass index

CABG = coronary artery bypass graft

CPR = cardiopulmonary resuscitation

eGFR = estimated glomerular filtration rate

IABP = intraaortic ballon pump

ICU = intensive care unit

OHCA = out-of-hospital cardiac arrest

PCI = percutaneous coronary intervention

SAPS II = Simplified Acute Physiology Score II

SAVE score = Survival after Veno-Arterial ECMO Score

VA-ECMO = venoarterial extracorporeal membrane oxygenation
